# Self-management behavior and fasting plasma glucose control in patients with type 2 diabetes mellitus over 60 years old: multiple effects of social support on quality of life

**DOI:** 10.1186/s12955-021-01881-y

**Published:** 2021-11-12

**Authors:** Xinye Qi, Jiao Xu, Guiying Chen, Huan Liu, Jingjing Liu, Jiahui Wang, Xin Zhang, Yanhua Hao, Qunhong Wu, Mingli Jiao

**Affiliations:** 1 Department of Health Policy, Health Management College, Harbin Medical University, 157 Baojian Road, Nangang District, Harbin, Heilongjiang China; 2 Department of Social Medicine, School of Public Health, Harbin Medical University, 157 Baojian Road, Nangang District, Harbin, Heilongjiang China; 3 Department of Cardiology, First Affiliated Hospital of Harbin Medical University, Harbin, Heilongjiang China

**Keywords:** Elderly, Hypoglycemia, Quality of life, Patient self-management, Social support

## Abstract

**Objective:**

Elderly patients with type 2 diabetes mellitus are highly vulnerable due to severe complications. However, there is a contradiction in the relationship between social support and quality of life, which warrants further exploration of the internal mechanism. This study assessed the quality of life and its interfering factors in this patient population.

**Methods:**

In total, 571 patients with type 2 diabetes mellitus over 60 years old were recruited from two community clinics in Heilongjiang Province, China. We collected data on health status, quality of life, self-management behavior, fasting plasma glucose (FPG) level, and social support. Structural equation modeling and the bootstrap method were used to analyze the data.

**Results:**

The average quality of life score was − 29.25 ± 24.41. Poorly scored domains of quality of life were “Psychological feeling” (− 8.67), “Activity” (− 6.36), and “Emotion” (− 6.12). Of the 571 patients, 65.32% had normal FPG, 9.8% had high-risk FPG, 15.94% had good self-management behavior, and 22.07% had poor social support. Significant correlations among social support, self-management behavior, FPG level, and quality of life were noted. A multiple mediator model revealed that social support influenced quality of life in three ways: (1) directly (c′ = 0.6831); (2) indirectly through self-management behavior (a1*b1 = 0.1773); and (3) indirectly through FPG control (a2*b2 = 0.1929). Self-management behavior influenced the quality of life directly and indirectly through FPG control.

**Conclusion:**

Improving self-management behavior and monitoring hypoglycemia should become priority targets for future intervention. Scheduled social support to self-management projects should be put into the standardized management procedure. Physicians should provide substantial and individualized support to the elderly patients with type 2 diabetes mellitus regarding medication, blood glucose monitoring, and physical exercise.

**Supplementary Information:**

The online version contains supplementary material available at 10.1186/s12955-021-01881-y.

## Introduction

The incidence of type 2 diabetes mellitus has been overgrowing due to lifestyle changes, urbanization, and aging. Between 2000 and 2016, there was a 5% increase in premature mortality from diabetes [1]. Type 2 diabetes mellitus accounts for 90% of diabetes cases worldwide [2], and the global prevalence of adult diabetes has increased dramatically from 4.7% in 1980 to 9.0% in 2014 [3] and 9.5% in 2019 [4]. In China, the percentages of adult patients with diabetes has increased from 0.67% in 1979, to 2.7% in 2002, to 11.6% in 2010 [5, 6], to 10.9% in 2013 [7], to 11.2–12.8% in 2017 [8, 9], and 10.9% in 2019 [10]. Sixty to eighty percent of the increment occurred in developing countries [11, 12], and 40% of patients with type 2 diabetes mellitus worldwide were the elderly [13]. The proportion of elderly patients with type 2 diabetes mellitus in China has increased dramatically from 10.2% in 2000 to 13.6% in 2006, 20.4% in 2007, 22.86% in 2010 [5, 6, 14], 34.1% in 2017 [15], and 35.5% in 2019 [10], which indicates a severe public health issue. Diabetes with severe chronic complications imposed a heavy economic burden on patients and decreased their quality of life [16]. With a gradually declining physical condition and increasingly poor income status, elderly diabetes patients may have difficulty self-managing their health, thus becoming a vulnerable population [17]. As important as biomedical markers, the quality of life of elderly patients with type 2 diabetes mellitus should receive more attention and prioritized care from medical staff and society [18].

Social support refers to an individual’s perception of spiritual or material support from family, friends, and other important relations. Excellent social support is the basis for improving the quality of life and played an essential role in relieving mental pressure, eliminating psychological obstacles, enhancing the effects of therapy, and optimizing the prognosis. Higher social support (e.g., family, friends, community) is linked to better outcomes in patients with diabetes [19]. The stress-buffering model [20] suggests that social support is related to outcomes due to its possible role in regulating stress function, and is a practical psychological resource in reducing stress and promoting health and well-being [21]. Also, the main-effect model of social support proposes that irrespective of whether individuals are under stress or not, social support resources encourage health-supporting behaviors and directly benefit health outcomes or wellness because it boosts overall well-being [22].

Self-management of chronic illness refers to individuals’ daily activities to keep their disease under control and minimize its impact on physical health status. World Health Organization proposed that anyone with a long-term health problem can address a challenging health-related situation by setting goals or guidelines for self-management [23]. The treatment of diabetes is complex and multidisciplinary [24]. Its three main goals are as follows: (1) to control complications; (2) to prevent hyperglycemia/hypoglycemia; and (3) to maintain a patient’s quality of life. A review claiming successful self-management is a crucial factor in the physical and psychological well-being of patients with diabetes [25]. Given the complexity of diabetes and its various complications, burdensome self-management activities such as daily diet, physical activity, blood glucose monitoring, and medication adherence, are essential [26] abilities of successful metabolic control to diabetes patients [27]. In recent years, the Chinese government has paid close attention to the management of diabetes, including diabetes screening and healthy lifestyle promotion, but little progress has been achieved. More than a quarter of patients with diabetes have poor self-management, and only 32–49% of patients have adequately controlled blood glucose levels [5, 28]. A meta-review of quantitative systematic reviews revealed that self-management did not improve other physiological targets of diabetes care rather than glycemic control, which may be caused by the narrow focus on glycemic control [29]. Thus, the long-term balance of blood glucose in patients with diabetes is clinically emphasized [30], which may lead to misinterpretation and hypoglycemia events during the treatment, resulting in the loss of quality of life.

Adequate blood glucose control does not only prevent and reduce the complications of diabetes but also decreases the probability and risk of hypoglycemia. Severe hypoglycemic events can cause unconsciousness, myocardial ischemia, hemiplegia, arrhythmias, myocardial infarction, cardiac failure, or even death [31] and are usually ignored [32]. Bramlage et al. found that the incidence of hypoglycemia increased with age [12.8% (> 75 years), 9.0% (< 60 years)] [33]. Additionally, a longitudinal study conducted by Lee et al. inferred that 28.3% of elderly with diabetes who had experienced a severe low blood sugar episode (Hypoglycemia) died within three years of the incident [34]. Elderly patients have difficulty perceiving hypoglycemia due to functional impairment of the nervous system, which lowers the blood glucose threshold sensitivity and increases severe hypoglycemia [35]. Therefore, avoiding hypoglycemia in elderly patients with type 2 diabetes should be a top priority.

The guideline for the management of diabetes mellitus in elderly in China (2021) recommended the need to carry out blood sugar self-monitoring to capture the occurrence of hypoglycemia events timely [36]. The occasional occurrence of hypoglycemia or abnormal blood glucose fluctuation in elderly patients with type 2 diabetes mellitus might have substantial, negative, and even severe clinical effects [37]. Despite the evolution of diabetes management technologies, blood glucose monitoring still plays an irreplaceable role in diabetes management [38]. Besides, an all-cause mortality analysis of the effect of abnormal fasting plasma glucose (FPG) control level on the Acute Myocardial Infarction revealed that increased and decreased FPG level at admission was a predictor factor to higher mortality rates [39]. In China, the rate of blood glucose self-monitoring is only 21.4%, and hypoglycemia occurs in 30% of elderly patients with type 2 diabetes mellitus. Carlene et al. found that each 1-mmol/l decrease of FPG was associated with a 21% lower risk of stroke and a 23% lower risk of ischemic heart disease [40]. Fang et al. carried out continuous glucose monitoring in elderly male patients with type 2 diabetes and revealed the significant relationship between FPG and nocturnal hypoglycemia [41]. Despite a large number of elderly population with diabetes, the association of self-management behavior and glycemic control is controversial [42, 43], and limited research in China investigating psychological and behavioral factors and their collective impacts on glycemic control. Therefore, FPG control is an important issue among elderly patients with diabetes, and self-management behavior may mediate the relationship between social support and glycemic control among type 2 diabetes patients.

While social support is usually conceptualized and perceived as a positive resource in chronic disease, it sometimes turns into a negative experience and may deteriorate health. Thus, disputes and inconsistent findings relate to the relationship between social support to quality of life of patients with diabetes [44]. According to Bandura’s social theory, factors such as social support are practical aspects in the incidence of the behavior [45]. Walker et al. explored the relationship of psychological and socioeconomic factors on diabetes self-care, and considered social support as one psychosocial factor associated with self-care behaviors [46]. Previous studies have documented that high social support can contribute to successful diabetes self-management [49, 50]. For example, a meta-analysis of 122 studies conducted by DiMatteo showed that self-management with medical regimens in patients with social support increases by 27% [47]. Poor social support to the elderly may lead to unrecognized complications, irregular treatment, and poor self-management behavior (e.g., diet, exercise, medication, blood glucose monitoring). In turn, poor self-management behavior may cause persistent hyperglycemia/hypoglycemia or glucose level fluctuation. A systematic review concluded that higher levels of social support are associated with improved clinical outcomes and the adaptation of beneficial lifestyle activities [48]; however, the role of social support in diabetes self-management and outcomes is not well understood [49, 50]. Young et al. argued that inadequate family and/or social support might cause suboptimal self-management behavior, indicating the need to consider monitoring the patients’ self-management behaviors and psychosocial factors [51]. Thus, we assumed that patient performance of self-management behavioral activities and psychosocial factors (e.g., social support) affected the patients’ clinical outcomes.

So far, most previous studies have focused on all age groups and not elderly diabetes patients, and the relationship between social support and quality of life is controversial, including the complex internal mechanism of multiple variables among elderly diabetes. Social support (e.g., family, peer support, caretakers) is considered as one of the psychosocial factors for self-management behavior, clinical outcomes [52, 53], and quality of life [51]. The American psychologist Baumeister et al. proposed the mechanism of Ego Depletion addressing self-control and active activities relay on the limited psychosocial resources [54], which varies from individuals [55]. Tang et al. considered social support as a psychosocial factor and indicated that perceived social support plays a vital role in the diabetes-specific quality of life and self-care behavior practices. Social support encompasses multiple dimensions that influence specific diabetes health-related outcomes and behaviors [56]. Therefore, perceived social support may be a remarkable predictor of self-care behavior and disease control in diabetes patients.

There is an urgent need to explore the psychological impact of self-management behavior and its impact on diabetes-specific quality of life and well-being, and the affection of self-management behavior on hypoglycemia or blood glucose indicators [46, 57]. Different theoretical perspectives provide clues for identifying the relevant psychosocial determinants of improving the quality of life in elderly diabetes patients (Additional file 1: eFigure1). According to the chronic care model, mobilize resources (e.g., social support, financial adequacy) provided by healthcare providers are needed to improve health outcomes and process parameters for elder diabetes patients [58, 59]. Meanwhile, the AADE7 Self-Care Behaviors ® (AADE7) framework also addressed learning, behavioral, clinical, and technology use effectively, improving the quality of life outcomes for diabetes, and achieving behavior change for better self-management behavior [60]. Based on the theory of the Chronic Care Model and the AADE7 Self-Care Behaviors ® (AADE7) framework, we hypothesized that the relationship between social support and quality of life would be explained, in part, by an indirect effect via diabetes self-management behavior and FPG control. The hypothesis was put forward that with control of these indirect paths, the direct relationship between social support and quality would be substantially reduced. Path analysis was conducted to evaluate all indirect pathways from social support to quality of life by inspecting the direction and magnitude of path coefficients. All the one-way paths were considered **(**Additional file 1: eFigure2). We examined the direct pathway from social support to quality of life (Arrow A). And we also investigated whether this relationship could be explained partially by indirect effects through diabetes self-management (Arrows B and C) or FPG level (Arrows D and E). Additionally, a double-mediator pathway from social support to quality of life by both self-management (primary mediator) and FPG level (secondary mediator; Arrows B, F, and E) was investigated.

## Methods

### Participants

Elderly patients with type 2 diabetes mellitus were recruited from the Jianhua community, Qiqihar City, China, between June and December 2012. The inclusion criteria were as follows: (a) a diagnosis of type 2 diabetes mellitus made by a physician at least one year before the study; (b) able to self-manage their health and (c) age > 60 years old. The exclusion criteria were patients with: (a) acute or chronic inflammatory disease; (b) cancer; and (c) type 1 diabetes mellitus. All participants signed a formal consent form before enrolment into this study.

### Procedures

Survey and standard investigation procedures were carried out to ensure the uniformity of data collection. First, chronic illness records of type 2 diabetes mellitus patients were reviewed, and their eligibility was evaluated. Second, elderly patients with type 2 diabetes mellitus were encouraged to participate in the program after explaining the purpose of the study. Third, appointments were scheduled for the participants to complete the questionnaire. At this point, the researchers elaborated on the purpose of the study and confirmed the patients' eligibility. Patients who agreed to participate in the study signed a formal consent form. The following variables were also collected: age, sex, race, education, income level, marital status, age at disease onset, duration of diabetes, FPG level, social support, self-management behavior, and quality of life.

### Measures

#### Perceived level of social support

The Multidimensional Scale of Perceived Social Support (MSPSS) developed by Zimet et al. [61] was used to measure the patients’ perception of the perceived availability and adequacy of emotional and instrumental social support (helping to make decisions, taking action, and so on) [62]. The overall psychometric properties of the MSPSS are strong. The scale contains 12 items evaluating three dimensions of support: family (including parents, children, and spouse [items 3, 4, 8, and 11]); friends (items 6, 7, 9, and 12); and other important relations (including neighbors and doctors [items 1, 2, 5, and 10]). Each item was scored on a 5-point scale ranging from 1 (strongly disagree) to 5 (strongly agree), with the total score ranging from 12 to 60 [63]. A higher score indicates better overall social support. In this study, scores >  = 12 but <  = 36 mean a low to moderate perceived social support, while scores > 36 but <  = 60 mean a high perceived social support [64, 65].

Previous studies have demonstrated the reliability of MSPSS, with Cronbach’s α ranging from 0.85 to 0.94 [61, 66, 67]. Test–retest reliability was evaluated over a 2–3-month interval (r = 0.72–0.85) [68]. In this study, Cronbach’s α was 0.855, 0.835, 0.841, and 0.929 for each subscale and the overall scale, respectively.

#### Quality of life

The Chinese version of the Adjusted Diabetes-specific Quality of Life Scale (CN-ADDQOL), was used after cultural adaptation and revision of the original scale [69]. The scale consists of 19 items and five dimensions: leisure activities (1 to 5), emotional feelings (6 to 9), psychological feelings (10 to 14), family living conditions (15 to 17), and diet (18, 19). The participants were requested to evaluate their actual situation and the importance of each item. For example, the question-for item 10 is, “If I would not have diabetes, what would my physical appearance be like?”; options were very good (− 3 points), good (− 2 points), satisfactory (− 1 point), the same as now (0 points), and worse than now (l point). For the importance of “my physical appearance,” options were very important (3 points), important (2 points), somewhat important (1 point), and not important at all (0 points). If the participant chose very good and very important, respectively, the score of this item was − 3*3 = − 9 [70, 71]. The total score ranged from − 171 to 57, with a higher score indicating a better quality of life [72, 73]. Cronbach’s α for the original scale was 0.81–0.941 [69, 74], and the comparative fitting index (CFI) of the structural equation model was 0.96 [75]. In this study, Cronbach’s α for CN-ADDQOL was 0.885.

#### Self-management behavior

A modified version of the Type 2 Diabetes Self-care Scale (2-DSCS), developed by Toobert et al. and Wang et al. to measure diabetes self-management behavior [76], was used in this study. The modified scale comprises 26 items encompassing six dimensions: diet (6 items), exercise (4 items), medication (3 items), blood glucose monitoring (4 items), foot care (5 items), and hypo/hyperglycemia (4 items). Items were scored from 1 (never) to 5 (always) [66, 77]. The total score ranged from 26 to 130, with a higher score indicating a higher level of self-management. The score index (score index = actual total score/possible highest score*100%) and the standard score (standard score = actual score/possible highest score*100) of each dimension were calculated to facilitate the comparison of data. A score index or standard score < 60% was considered poor, 60–80% was considered medium, and > 80% was considered good [77]. Cronbach’s α for 2-DSCS was 0.82–0.88, and test–retest reliability was good, at 0.92–0.96 [78, 79]. In this study, Cronbach’s α for each of the six dimensions was 0.919, 0.891, 0.863, 0.836, 0.783, and 0.844, respectively; the total scale had good internal consistency (Cronbach’s α = 0.880).

#### FPG level

FPG is an essential indicator of hypoglycemia and hyperglycemia. Values > 3.1 mmol/L are considered relatively safe (the occurrence of hypoglycemia is improbable). While the control of FPG should not be too strict to avoid the possibility of a hypoglycemia crisis. When hypoglycemia occurs and remains unnoticed for a certain period, irreversible body injury may occur [80]. Chinese guidelines for diabetes prevention do not recommend strict blood glucose control in elderly patients with type 2 diabetes mellitus [24]. The Chinese guidelines for Diabetes Prevention and Control (2017 edition) recommends the following FPG levels explicitly: > 5.0 and <  = 7.2 mmol/L for elderly patients with type 2 diabetes mellitus living with more minor chronic disease, and complete cognitive and functional status; > 5.0 and <  = 8.3 mmol/L for elderly patients with type 2 diabetes mellitus living with complicated health status. Besides, an FPG level above 16.7 mmol/L is defined as severe hyperglycemia [24, 36]. On the morning of the scheduled appointment, a blood sample was obtained from each patient. Twelve-hour FPG levels were assessed according to World Health Organization (WHO) standardized fingertip pricked test procedures using calibrated blood glucose meters and reagent strips [81]. In this study, an FPG level of 5.0–8.3 mmol/L was defined as successful FPG control.

### Data analysis

Statistical analyses were performed using SPSS, version 17.0 (IBM, Armonk, NY, USA) and structural equation modeling (SEM) was conducted using AMOS 17.0 (IBM). Missing data were imputed by expectation maximization (EM) using SPSS missing value analysis. Missing data for quality of life, social support, and self-management behavior were 0–4.2, 0–2.5, and 0–3.5%, respectively. Descriptive statistics were used to summarize patient characteristics and measured variables.

The SEM was deemed suitable for developing a model to explain relationships among the study variables based on the variance/covariance matrix using maximum likelihood estimation. The hypothesized model was evaluated using the following multiple criteria of goodness-of-fit: a) χ^2^/df ≤ 2; b) CFI > 0.95 [78]; c) goodness-of-fit index (GFI) > 0.90; d) normed-fit index (NFI) > 0.90; and e) root mean square error of approximation (RMSEA) < 0.06. This analytical approach allows for sequential examination of two mediators while simultaneously testing the indirect effects of each mediator independently [82]. Variables with non-significant factor loadings were deleted from the structural equation model. Chi-square difference tests and the Akaike information criterion were used to compare the alternate and theoretical models [83]. A two-tailed *p*-value of 0.05 indicates statistical significance. The bootstrap method was used to test the multiple mediating effects of the hypothesized model [84, 85]. All specific and conditional indirect effects were subjected to follow-up bootstrap analyses [85].

## Results

### Patient characteristics

A total of 571 elderly patients with a mean duration of type 2 diabetes mellitus of 8.23 ± 6.85 years were enrolled in this study. Their general characteristics and scores of the quality of life are shown in Table 1. Among the 571 patients, 48.3% feared hypoglycemia occurrence; 22.07% had poor-to-moderate social support; 33.62% had poor self-management behavior while 15.94% had good self-management, and 30.99% had poorly controlled FPG levels (≥ 8.3 mmol/L), 9.98% had high-risk FPG level, while 65.32% had successful FPG control (5.0–8.3 mmol/L). Patients with low to moderate social support and poor self-management behavior had the lowest quality of life scores, followed by those with FPG > 16.7 mmol/L and those who never engaged in physical exercise. The constituent ratios for each item of the MSPSS and 2-DSCS are shown in Additional file 1: eFigure3 and eFigure4, respectively. The specific scores for each dimension of self-management behavior and social support are shown in Additional file 1: eTable1. Among the patients with poor self-management behavior, 60.8% had poor exercise management, 50.1% had poor blood glucose monitoring ability, and 40.8% had poor FPG control over hypo/hyperglycemia. More than 20% of those patients reported poor social support. Quality of life, social support, and self-management behavior data are summarized in Additional file 1: eTable2.

**Table 1:** Descriptive statistics for the total sample and scores of the quality of life in the elderly patients with type 2 diabetes mellitus with different demographic characteristics (n = 571)

Variable	n (%)	Quality of life score (mean ± SD)
Age, years		
> = 60, < 65	147 (25.74)	− **33.16 ± 26.11**
> = 65, < 70	129 (22.59)	− **28.06 ± 22.84**
> = 70, < 75	159 (27.85)	− **29.27 ± 25.49**
> = 75, < 80	89 (15.59)	− 26.15 ± 21.47
> = 80	47 (8.23)	− 26.05 ± 19.81
Sex		
Men	191 (33.45)	− 28.50 ± 23.23
Women	380 (66.55)	− **29.62 ± 24.56**
Education		
< High school	309 (54.12)	− 27.60 ± 21.41
High school	207 (36.25)	− **32.36 ± 26.27**
> High school	55 (9.63)	− 26.74 ± 28.83
Marital status		
Single	6 (1.05)	− **29.22 ± 18.13**
Married	465 (81.44)	− **29.70 ± 25.06**
Divorced	18 (3.15)	− 26.89 ± 19.13
Widowed	82 (14.36)	− 27.19 ± 19.72
Income group		
< 1000RMB	127(22.2)	− 30.33 ± 23.66
> = 1000, < 3000RMB	406(71.1)	− 28.89 ± 23.57
> = 3000RMB	38(6.7)	− 29.73 ± 29.08
Frequency of physical exercise		
Never	23 (4.03)	− **34.77 ± 37.13**
Occasionally	44 (7.71)	− **32.69 ± 27.12**
Irregular	183 (32.05)	− **30.52 ± 23.91**
Frequently	203 (35.54)	− 28.64 ± 25.16
All the time	118 (20.67)	− 25.95 ± 19.17
Level of social support		
Low to moderate	126 (22.07)	− **44.66 ± 26.03**
High	445 (77.93)	− 24.88 ± 22.09
Self-management behavior^#^		
Bad	192 (33.62)	− **40.71 ± 26.35**
Medium	288 (50.44)	− 25.39 ± 21.72
Good	91 (15.94)	− 16.38 ± 17.31
FPG, mmol/L		
> 3.9, < = 5.0	57 (9.98)	− 24.66 ± 19.69
> 5.0, < = 8.3	373 (65.32)	− 28.14 ± 24.31
> 8.3, < = 16.7	137 (23.99)	− **33.94 ± 24.40**
> 16.7	4 (0.70)	− **36.83 ± 36.51**
PBG, mmol/L		
≤ 7.8	76 (13.31)	− **28.66 ± 25.96**
> 7.8, < = 11.1	213 (37.30)	− 25.42 ± 20.59
> 11.1	282 (49.39)	− **32.29 ± 25.67**
Duration of diabetes, years		
≤ 1	95 (16.64)	− 21.84 ± 20.76
> 1, < = 3	92 (16.11)	− 25.80 ± 21.85
> 3, < = 5	65 (11.38)	− **29.17 ± 21.29**
> 5, < = 10	149 (26.10)	− **32.12 ± 25.42**
> 10	170 (29.77)	− **32.76 ± 25.88**
Mean ± SD	8.23 ± 6.85	− 29.25 ± 24.41
Complications		
Yes	472 (82.66)	− **31.07 ± 25.47**
No	99 (17.34)	− 20.23 ± 12.35

The bold font indicates poor quality of life; ^#^ based on the score index and the standard score

FPG = fasting plasma glucose; PBG = postprandial blood glucose; SD = standard deviation

### Preliminary analysis: bivariate analysis

The results of the bivariate analysis are shown in Additional file 1: eTable 2. All correlations were significant. Tolerance values ranged from 0.693 to 0.804, and variance inflation factor (VIF) values changed from 1.244 to 1.443. Further, case analysis revealed no evidence of outliers. The results of correlation analysis provided the basis for testing the mediation effect.

### Multiple mediation analysis

Baron and Kenny’s mediation effect testing procedure was used to verify the hypothesis model. Model 1 was a theoretical model depicting each path between social support and quality of life with mediators (self-management behavior and FPG). Three competitive models (Models 2, 3, and 4) and one alternative model (Model 5) were also analyzed.

**Fig. 1:**
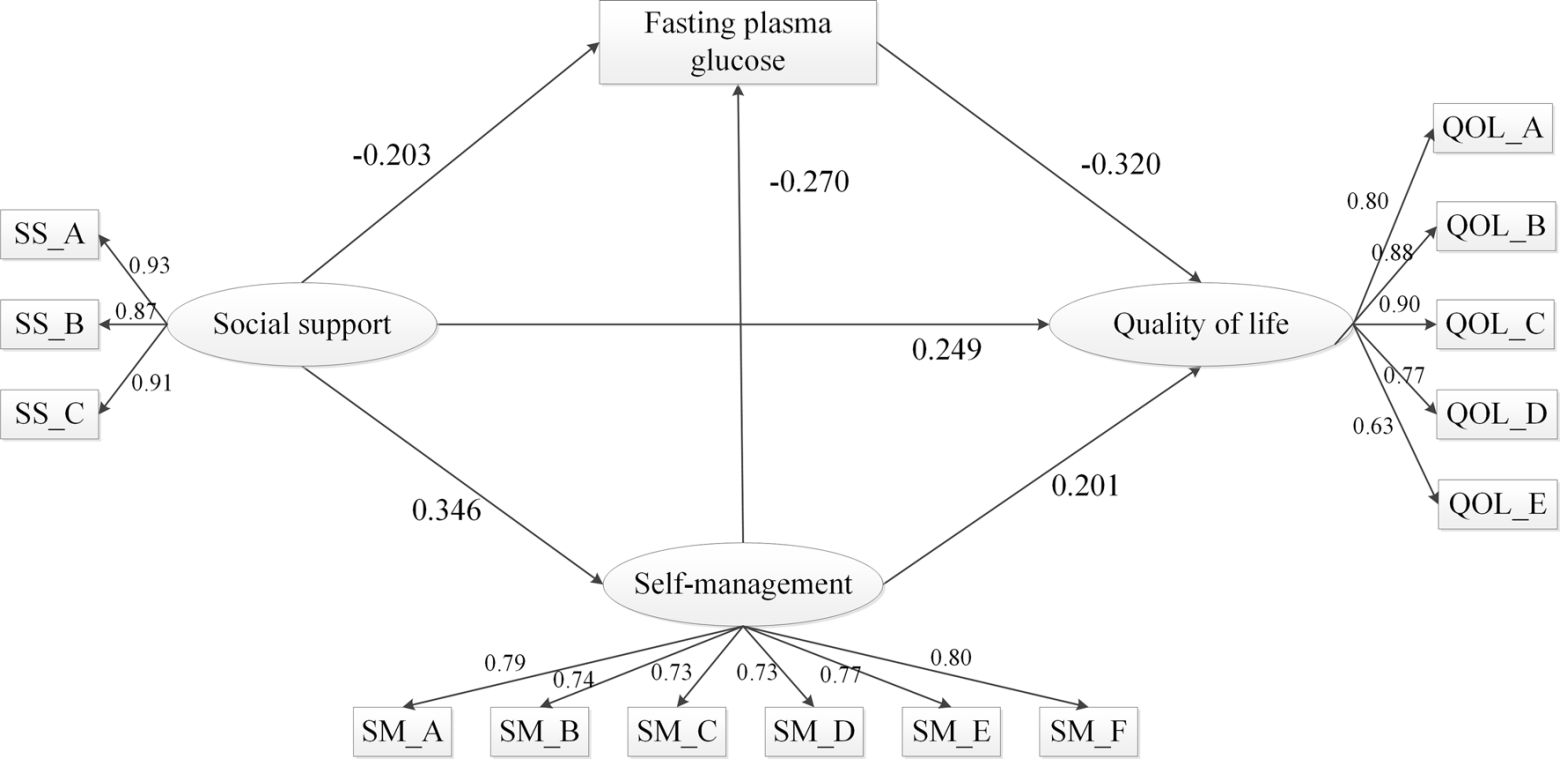
Multiple mediation models of social support and quality of life. SM_A = diet control management; SM_B = exercise management; SM_C = medication management; SM_D = blood glucose monitoring management; SM_E = foot care; SM_F = hypoglycemic/hyperglycemia management; QOL_A = quality of life, activity domain; QOL_B = quality of life, emotion domain; QOL_C = quality of life, psychological feeling domain; QOL_D = quality of life, family burden domain; QOL_E = quality of life, diet domain; SS_A = family support; SS_B = friends support; SS_C = support from others

The fit indices of Models 1, 2, 3, 4, and 5 are shown in Table 2. Models 2, 3, 4, 5 were compared against Models 1, and the comparisons indicated that Δ**χ**^**2**^ was significantly difference of Model 2, 3, 4, 5 with Model 1 (all *p* value < 0.001). Additionally, Model 1 showed a better fit than other models and all paths were significant (Additional file 1: eTable3). Therefore, Model 1 was considered the best model to match the observation data (Fig. 1, The final mediation model). Paths from social support to self-management (a1 = 0.329, *p* < 0.001), FPG level (a2 = − 0.186, *p* < 0.001), and quality of life (c′ = 0.496, *p* < 0.001) were significant. The path coefficients from self-management activity to FPG level (a3 = − 0.260, *p* < 0.001) and quality of life (b1 = 0.422, *p* < 0.001) were significant. The path coefficient from FPG level to quality of life (b2 = 0.697, *p* < 0.001) was also significant.

**Table 2:** Comparison of different structural equation models

Model Description	χ^2^	DF	GFI	NFI	CFI	IFI	RMSEA	Δχ^2^
Model1: Hypothetical mediation model	238.01	85	0.948	0.956	0.971	0.971	0.056	
Model2: Deletion of path from SM to QOL	259.05	86	0.943	0.952	0.968	0.968	0.059	21.04***
Model3: Deletion of path from SM to QOL and from SM to FPG	280.17	87	0.939	0.948	0.964	0.964	0.062	42.16***
Model4: Deletion of path from SM to FPG	273.97	86	0.940	0.950	0.965	0.965	0.062	35.96***
Model5: Alternative model: SS, SM, and FPG directly affect QOL	384.57	88	0.914	0.929	0.944	0.945	0.077	146.56***

DF = degrees of freedom; GFI = goodness-of-fit index; NFI = normed-fit index; CFI = comparative fit index; IFI = incremental fit index; RMSEA = root mean square error of approximation; SM = self-management behavior; SS = social support; QOL = quality of life; FPG = fasting plasma glucose

***p < 0.001

### Significance test of the mediation effect

The estimates and bootstrapped 95% confidence intervals (CIs) of the indirect effects were the paths verified for mediation (Table 3). The finalized structural model (Fig. 1) revealed that the paths from social support to quality of life through self-management and FPG levels were significant. The results showed that the 95% CIs of the indirect effects differed significantly from zero, and the mediating effects had statistical significance (p < 0.05). Point estimates for indirect effects and 95% bias-corrected CIs for multiple mediation analyses revealed that self-management and FPG level were mediators in the path between social support and quality of life.

**Table 3:** Bootstrap test results (indirect effects of X on Y)

Mediation path	Effect	Boot SE	*p*	95% CI, lower limit	95% CI, upper limit
SS → SM → QOL (a1*b1)	0.1773	0.0410	< 0.0001	0.1044	0.2688
SS → SM → FPG → QOL (a1*a3*b2)	0.0770	0.0187	< 0.0001	0.0464	0.1236
SS → FPG → QOL (a2*b2)	0.1929	0.0430	< 0.0001	0.1165	0.2831
SS → QOL (c—c′)	0.4473	0.0555	< 0.0001	0.3425	0.5645
SS → QOL (c’)	0.6831	0.1096	< 0.0001	0.4679	0.8982
c	1.1304				

SS was the independent variable (X), SM (M1) and FPG (M2) were the mediators, and QOL (Y) was the outcome. a1*b1 and a2*b2 = indirect effects of X on Y through M1 and M2; a1*a3*b2 = indirect effect of X on Y through M1 and M2; and c′ = direct effect of X on Y; c = the total effect of X on Y (a1*b1 + a1*a3*b2 + a2*b2 + c′). The 95% CIs for indirect effects were obtained by bootstrapping with 5,000 resamples. 95% CI, lower limit = lower bound of a 95% CI; 95% CI, upper limit = upper bound of a 95% CI. →  = “affects.”

CI = confidence interval; FPG = fasting plasma glucose; SE = standardized estimate; SM = self-management behavior; SS = social support; QOL = quality of life

The indirect effect on SS → SM → QOL path (a1*b1) was 0.1773 (95% CI [0.1044, 0.2688], *p* < 0.0001), taking up 15.69% of the total effect (a1*b1/c) and 39.64% of the total indirect effect (a1*b1/c–c′). The indirect effect on SS → FPG → QOL path (a2*b2) was 0.1929 (95% CI [0.1165, 0.2831], *p* < 0.0001), taking up 17.07% of the total effect (a2*b2/c) and 43.13% of the total indirect effect (a2*b2/c–c′). The indirect effect on SS → SM → FPG → QOL path (a1*a3*b2) was 0.0770 (95% CI [0.0464, 0.1236], *p* < 0.0001), taking up 6.81% of the total effect (a1*a3*b2/c) and 17.21% of the total indirect effect (a1*a3*b2/c–c′).

In the relationship of social support to quality, the direct effect of social support on quality of life was 0.6831 (95% CI [0.4679, 0.8982], *p* < 0.0001); the indirect effect of social support on quality of life (controlling for the mediators) was 0.4473 (95% CI [0.3425, 0.5645], p < 0.0001), which accounted for 39.57% (c–c′/c) of the total effect of social support on quality of life (1.1304).

## Discussion

This study underscores the critical roles of social support, self-management behaviors, and FPG control in the quality of life among elderly patients with diabetes. The results revealed that social support directly influences the quality of life and indirectly predicted quality of life through self-management behaviors and FPG control level. It is also shown that self-management behaviors, directly and indirectly, influenced the horizontal quality of life through the FPG control level. Furthermore, a novel chain-mediation model revealed that self-management behaviors and FPG control level mediated the relationship between elderly diabetes patients' social support and quality of life. This study elucidated a complex internal relationship among social support, FPG level, self-management behavior, and quality of life in elderly patients with diabetes and provided a detailed and in-depth explanation of the processes and mechanism of how perceived social support affects quality of life among elderly diabetes patients. Based on these findings, general practitioners and physicians highly recommended finding ways to accomplish and facilitate social support intervention protocol involving optimum FPG control by strengthening self-management behavior targeting the eventual improvement of the quality of life of elderly diabetes.

Elderly patients with type 2 diabetes mellitus had a moderate quality of life (− 29.25 ± 24.41), which was lower than reported by Kan et al. (− 13.57 ± 7.68 to − 11.25 ± 7.18) [86]; poorly scored dimensions of quality of life were “Psychological feeling” (− 8.67), “Activity (− 6.36),” and “Emotion” (− 6.12). At the same time, 82.66% of patients reported complications, indicating that the quality of life in elderly patients with type 2 diabetes mellitus in China is poor [74], which was consistent with the findings of previous studies [24]. Interestingly, we found that the group with low social support (score = − 44.66) and that with poor self-management behavior (score = − 40.71) had scores that were 43.66% and 39.71% lower than the average (score = − 29.25), respectively. Mohebi et al. inferred that social support significantly reduced with increased age and duration of diabetes [26]. With persistent and disease fluctuations and complications (82.66%), this could lead to poor quality of life. Their average scores were notably inferior in the psychological, activity, and emotional domains. Patients felt boresome, psychological and emotional fatigue, and more easily troublesome to talk to or seek help from their family members or friends, resulting in their poor use of support. Elderly patients with type 2 diabetes mellitus may experience inadequate social support and poor self-management, affecting the quality of life. Therefore, it is necessary to focus on the support of elderly diabetes patients.

### Mediating effect of self-management behavior

Mediation analysis results indicate that self-management behavior played a mediation role in the influence of social support on quality of life (SS → SM → QOL). Al-Dwaikat et al. claimed that self-management did not mediate the relationships between social support dimensions and their health outcomes [87], which is not consistent with the results in this study. The findings in this study suggested that self-management behaviors were a significant mediator in the association between social support and quality of life, which highlight the importance of implementing necessary social support to promote effective diabetes self-management behavior to achieve better health outcomes among elderly patients with type 2 diabetes.

Diabetes is a disease requiring long-term treatment, requiring patients to control their diet and self-monitor their blood glucose. Therefore, self-management of diabetes is of vital importance. Lee et al. used the theory of planned behavior, confirming that diabetes patients engaged in self-management education incorporate behavioral and psychosocial strategies (e.g., social support) with better diabetes outcomes [88]. Stopford et al. indicated that good diabetic health may not be sustainable because psychosocial factors hinder the best practice of self-management of diabetes [50]. Thus, the main support sources are important in the health care process [89]. In this study, the standard score of self-management behavior was 66.32 ± 13.47, lower than Lei et al. (78.94 ± 17.76 ~ 80.62 ± 17.77) [90]. The proportion of patients with reasonable diet control, regular exercise, medication management, blood glucose monitoring, foot care, and hyperglycemia/hypoglycemia management was 16.1%, 5.8%, 38.2%, 19.4%, 20.8%, and 31.3%, respectively. These results indicated that physical exercise and blood glucose monitoring engagement was weak in elderly patients with type 2 diabetes mellitus [69]. A systematic review evidenced that family support improved self-management behaviors and health outcomes in uncontrolled glycemia diabetes, Which indicating family engagement self-management education helps improve diabetes care activity [91]. It is necessary to highlight that failing to initiate personal actions and actions involving the family and the health care system will make the individual attempt to manage the disease the leading risk factor for experimenting with poor quality of life [92]. This study suggests that physicians should pay attention to improving the quality of life of elderly diabetes patients and pay attention to the self-management behaviors achievement of elderly diabetes patients. Practices (eg., education, information) can promote social support and guide elderly diabetes patients to the aspects and standards or methods they need for disease management.

### Mediating effect of FPG control

The indirect effect of perceived social support on QOL through FPG control suggesting that FPG control within the guidance range can play a critical mediation role in affecting the relationship between perceived social support and QOL. A systematic review of controlled intervention studies argued that prior studies on social support are not associated with better glycemic control [62], consistent with Chew et al., [49]. On the contrary, with previous reports [93, 94], this finding indicates that good perceived social support enables elderly diabetes patients to control FPG at an ideal level, which will benefit the quality of life of elderly diabetes patients (SS → FPG → QOL). Therefore, psychosocial factors are essential for FPG control.

The main-effect model of social support proposes that social resources have a beneficial effect irrespective of being under stress or not [22]. As a chronic distress exposure and stress [95], low perceived social support was associated with physiological alterations (e.g., activate the hypothalamic–pituitary–adrenal axis and sympathetic nervous system), which contributes to insulin resistance and poorer diabetes-related health [96]. Perceived social support was positively related to the release of oxytocin (a neuropeptide that relaxes individuals) [97]. A longitudinal study has revealed a positive association between baseline overall decline slope of cortisol (a stress hormone) and FPG change, which indicates that cortisol plays a detrimental role in the contribution to blood glycemia among diabetes patients [98]. Hooker et al. also highlighted that supportive relationships were essential protective factors to decrease high cortisol when the individual’s subjective socioeconomic status is low [99]. Research suggests that high social support has buffering effects that may be mediated through increased oxytocin concentrations, suggesting that oxytocin may be implicated in reducing free cortisol levels that increase during stressful events [97]. Therefore, chronic stress (e.g., poor perceived social support) and endocrine stress response (e.g., high cortisol, low oxytocin) are significantly related to insulin resistance and diabetes mellitus [100]. Thus, the relationship between perceived social support and FPG control level exists.

This study found that 65.32% of elderly diabetes patients’ FPG was under control, which was consistent with the findings of previous studies [101]. Many patients measure only their blood glucose when they are not feeling well. However, the recurrent fluctuation and variability of FPG will cause an abnormal increase in sympathetic nerve excitability and the increase of all-cause mortality and cardiovascular disease mortality [102, 103]. Notably, strict FPG control might be dangerous to multi-vulnerable patients due to hypoglycemia, dysfunctional osmolality, and consequences including death [104, 105]. Thus, FPG control should be listed as a priority target for intervention. Additionally, Zhang et al. also evidenced that hypoglycemia influenced patients >  = 65 years in diverse treatment pattern models [106]. A continuous blood monitoring study pointed that 93% of hypoglycemia events were not discovered among elder patients >  = 75 years [107]. Therefore, given the knowledge of the significant association between FPG and mortality/complications, special attention should be paid to fasting glucose monitoring. As Seaquist et al. suggested, it is necessary to emphasize individual management (e.g., education, diet, exercise, medicine adjustment, blood monitoring) to avoid excessive blood glucose control [108].

Lee et al. addressed the buffer effect of social support and revealed that adults with low autonomy support from family health supporters might be at risk for poor glycemic control [109]. Insufficient social support might, in turn, exert additional impact on FPG, and causing elderly diabetes patients more difficulty in keeping regular monitoring of blood glucose. However, the long-term cost of regularly self-monitoring blood glucose may also be very high for elderly diabetes patients. Yao et al. revealed a low frequency of blood glucose monitoring among patients with type 2 diabetes mellitus in China and recommending that educational and financial support increase blood glucose monitoring frequency in diabetes patients, especially patients with low socioeconomic status [110]. In this study, 22.2% of participants self-reported income were lower than RMB 1000. Therefore, nurses and physicians should address social support-based intervention protocols by mobilizing both external support (resources for regular monitoring) and subjective support (actions for regular monitoring) to achieve the monitoring target of FPG [111].

### Chain-mediating effect of self-management behaviors and FPG control

A notable finding of this study was the chain-mediating effect of self-management and FPG level in the relationship between social support and quality of life (SS → SM → FPG → QOL). This model illustrated that self-management behaviors acted as a mediator between social support and FPG control. FPG control mediated the relationship between self-management behavior and quality of life, indicating that the indirect effect of self-management behavior on quality of life through FPG was significant. Elderly diabetes patients in the present study who reported receiving more excellent support resources for disease management reported better self-management behaviors, which, in turn, affected the quality of life indirectly through the FPG control level. This finding demonstrated that self-management is essential to maintain ideal FPG levels, and poor FPG control can negatively impact their QOL. This finding was consistent with previously reported conclusions [112] and confirmed our hypothesis. One possible explanation is that self-monitoring of blood glucose is an integral part of diabetes treatment. Physicians need to formulate a hypoglycemic program for patients and a necessary reference for patients to observe blood glucose changes (hyperglycemia/hypoglycemia). Poor self-management behavior might result in non-timely monitoring of the FPG level, which, in turn, might reduce the quality of life.

Glycemic control is partly dependent upon the regular completion of several self-management behaviors, including exercise, dietary modification, foot care, self-monitoring of blood glucose, and medication adherence. A one-point score increase on the diabetes self-management scale leads to a 5% drop in the risk for suboptimal glycemic control [43], meaning that self-management plays an essential role in controlling glycemic. And it is necessary to enhance self-management in elderly patients with type 2 diabetes mellitus. Pilcher et al. defined social support as a self-control resource [113]. As posited in the Ego Depletion theory, poor self-management behavior is due to the lack of self-control resources of patients themselves, which is the root cause of management failure [114]. This study showed that only 15.94% of patients had high-quality self-management, while 84.06% had moderate or poor self-management; moreover, more than 20% of patients reported inadequate social support. The study found that elderly patients with type 2 diabetes mellitus could not self-manage their health, including physical exercise and diet, which contributed to poor quality of life. Therefore, it is necessary to strengthen the self-management behaviors and social support of elderly diabetes patients.

Reviews evidenced that the significant relationship of interventions (eg., exercise, diet) reduces poor FPG level and improves the quality of life [115, 116]. In line with the Guidance of the International Diabetes Federation (2014), individual blood sugar monitoring plans are urged to self-management arrangement [117]. Wang et al. found that 27.5% reported performing self-monitoring of blood glucose with the guidance of the Chinese Diabetes Society (2007) [118]. A nationally representative cross-sectional study of individual-level data in 680,102 adults from 55 low-income and middle-income countries revealed that only 4.6% of individuals with diabetes self-reported meeting the recommendation, diet counseling (32.2%), and exercise counseling (28.2%); and fewer than 10% of diabetes in developing countries received comprehensive diabetes treatment with guideline [119]. In this study, the top three self-management dimensions with poor score index were exercise (60.8%), blood glucose monitoring (50.1%), and hyperglycemia/hypoglycemia management (40.8%). To elderly diabetes patients, more self-management behavior will generate better-performed, goal-oriented effectiveness of FPG control. Therefore, health professionals and therapists should attempt to use self-care training methods and other training and therapeutic approaches to improve quality of life and self-care and reduce blood sugar, especially in the elderly with no self-care behaviors [120].

Lack of social support regarding diet, exercise, blood monitoring, et al., are significant barriers to self-management [51]. In this study, the total social support score in elderly patients with type 2 diabetes mellitus was 43.40 ± 8.41; family support scored the highest (14.75 ± 2.99) in the three dimensions of perceived social support, indicating that the main source of support was the patient’s family. According to the weak tie and strong tie theories [121], supports supplied by family or friends were regarded as strong ties [89], which is consistent with this study. Further analysis revealed that only 58.4% to 67.1% of patients received specific social support. Moreover, in this study, the proportion of patients who could not obtain sufficient support from family, friends, and essential others was 21.4%, 24.0%, and 25.4%, respectively; it indicated that community nurses and physicians might not be providing social support to elderly diabetes patients. Given the particularity of medical service demand of diabetes, despite physicians, nurses, communities, etc., were considered as weak ties in populations facing a threat [122], and other important support subjects (e.g., physicians, nurses) urgently needed to elder diabetes. Mohebi et al. emphasized the importance of patient-family communication [26]. The above study demonstrated that social support available from doctors, family, and friends was a significant potential resource for diabetes interventions, prioritizing attention. There is an urgent need to explore how to mobilize more actively, timely, persistent, and more substantial social support from families and society.

The Chronic Care Model and the AADE7 Self-Care Behaviors ® (AADE7) framework addressed social support and other factors that facilitate behavior modification [46, 123]. And it indicated that health care practitioners and future interventions are needed to improve individuals’ diabetes management behaviors (e.g., nutrition, exercise) [124, 125], with the ultimate goal of promoting glycemic control. Therefore, a fundamental translation of a collaborative-feedback partnership (e.g., family, friends, communities, nurses, and physician) of social support [126, 127] and regularly assessment [51] is critically needed, to comprehensively guidance target at the weakest areas of self-management. Theory-guided practice models [60, 128] practices including medication (dosing, frequency, and titration), self-monitoring blood glucose; food intake/eating patterns; and regular physical activity provided by the collaborative-feedback partnership are also needed to facilitate self-management behavior during routine appointments [51, 129].

### Limitations of research

This study had a few limitations. First, as a cross-sectional study, the relationships between variables were only correlative, and causal relationships could not be established. It’s also a pity that we didn’t measure the glycemia variability. Second, this study was performed in Heilongjiang Province, and the patients were recruited from city communities. Therefore, the findings of this study might differ from those of rural areas. Third, the MSPSS and 2-DSCS were revised in this study. Hence, the reliability of the scales requires further validation, despite good internal consistency. Fourth, this study has focused on the relationship between perceived social support and quality of life in elder diabetes, while sensitive information referring to psychological such as depression or psychological distress was not considered in the investigation. Thus, further research regarding psychological (e.g., depression or psychological distress) of health need to be addressed. Lastly, this study focused on perceived social support by elderly patients. Actual social support was not analyzed. With the development and abundance of social support resources, a specific social support scale for elderly patients with type 2 diabetes mellitus should be developed. In this study, some patients had difficulty in understanding some questions from the CN-ADDQOL, for the scale needs further modification. Further studies with larger, more diverse samples and more variables such as actual social support and specific self-management are needed.

## Conclusion

In conclusion, this study explored the underlying mechanisms between social support and quality of life among Chinese elderly patients with diabetes, which contributed to deepening the theoretical research on the quality of life by extending social support/self-management application to the quality of life. Elderly patients with type 2 diabetes mellitus had poor quality of life, fasting blood glucose control, and self-management. Our study identified four critical pathways constituting a complicated, interwoven network contributing to poor quality of life in elderly patients with type 2 diabetes mellitus. It also revealed the internal mechanism between critical variables of social support, self-management behavior, FPG level, and quality of life. The following vital interconnected paths were identified: SS → SM → QOL, SS → FPG → QOL, SS → SM → FPG → QOL, and SS → QOL.

Both social support and self-management behavior should be priority targets for future intervention. Particular attention should be paid to the quality of life and hypoglycemia in elderly patients with type 2 diabetes mellitus. These factors should be taken into consideration when developing personalized treatment and standardized management procedures.

## Supplementary Information

**Additional file 1**. Supplementary data of the conceptual and theoretical explanation and details of social support, self-management in this study.

## Data Availability

Not applicable.
