# Supplementary material for: Self-management behavior and fasting plasma glucose control in patients with type 2 diabetes mellitus over 60 years old: multiple effects of social support on quality of life
Source: Health Qual Life Outcomes. 2021 Nov 12;19:254. doi: 10.1186/s12955-021-01881-y (PMC8588678; doi:10.1186/s12955-021-01881-y)
Supplement: Supplementary file 1 — Additional file 1. Supplementary data of the conceptual and theoretical explanation and details of social support, self-management in this study. [file 12955_2021_1881_MOESM1_ESM.docx]

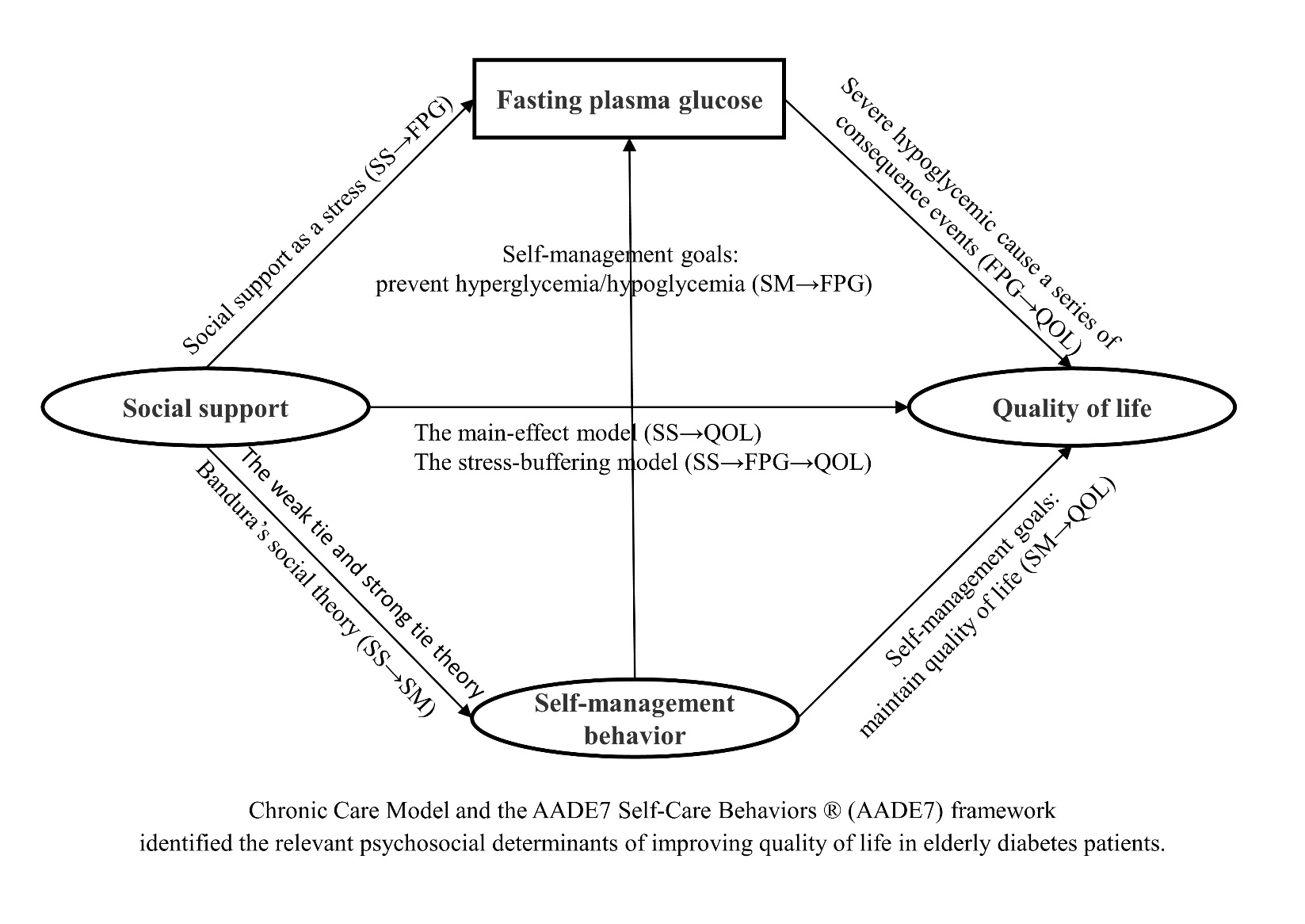


**efigure 1.** **The conceptual and theoretical explanation of the association between SS, SM, FPG, and QOL in elderly type 2 diabetes patients.**


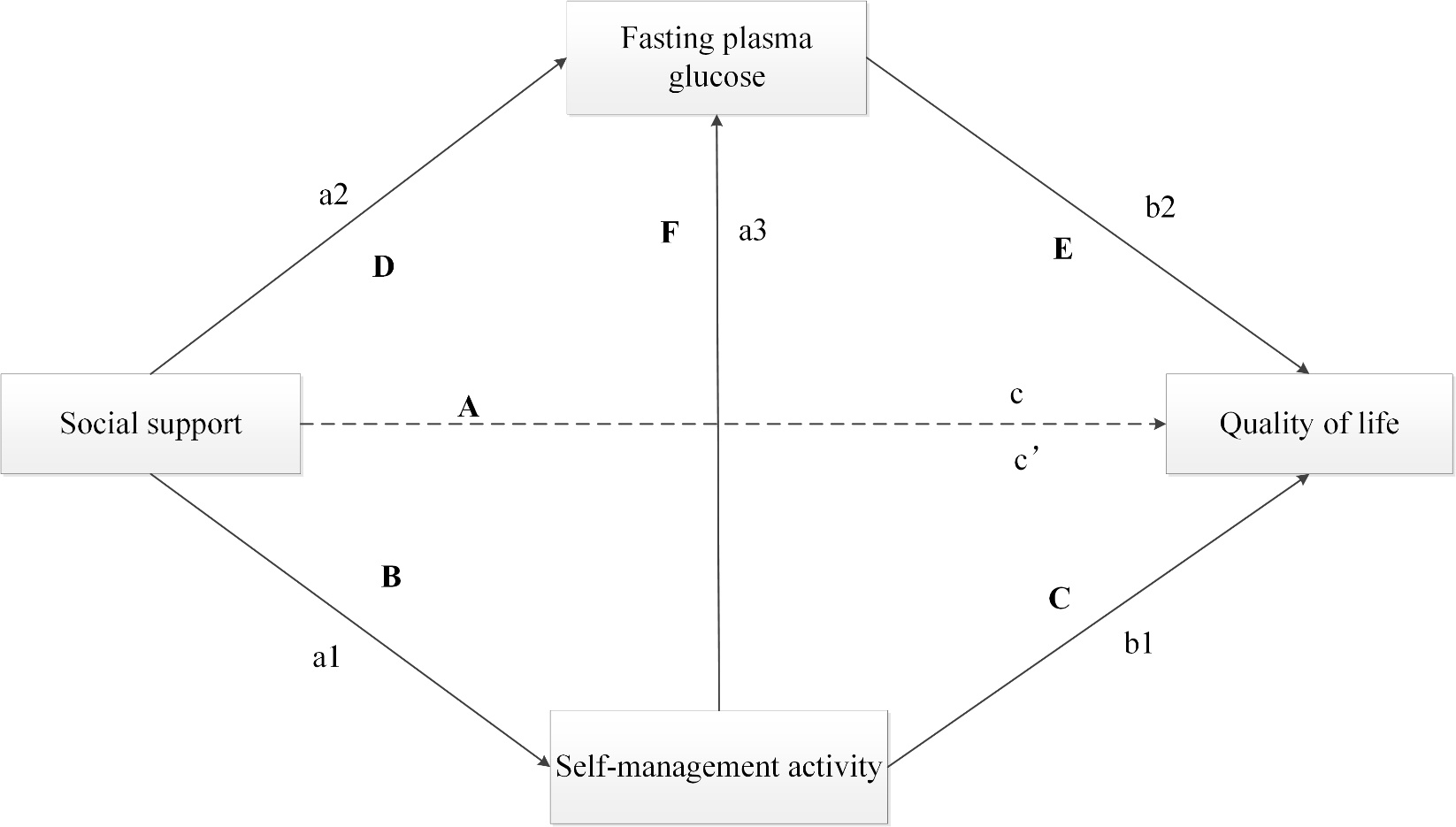


**eFigure 2**. **The conceptual path model for the affection of social support to quality of life;** In a simple mediation model, the impact of X on Y is considered a total effect (path c), interpreted as the expected amount by which two cases that differ by one unit on X are expected to differ on Y, which may occur directly or indirectly. Bias-corrected confidence intervals that did not include 0 were considered significant for the indirect effect of the X on Y was a mediator. The direct effect of X (path c′) is interpreted as the part of the effect of X on Y that is independent of the pathway through M. The indirect effect (a*b) is interpreted as the amount by which two cases who differ by one unit on X are expected to differ on Y through X’s effect on M, which in turn affects Y. This is the test of mediation (the effect of X on Y through M) or the difference between the total and direct effects. a1 and a2 = specific indirect effects of X on M1 and M2; a3 = specific indirect effect of M1 on M2; b1 and b2 = specific indirect effects of M1 and M2 on Y; c′ = direct effect of X on Y controlling for M1 and M2; a1*b1 and a2*b2 = specific indirect effects of M1 and M2; a1*a3*b2 = conditional indirect effect of M1 and M2.

c´ = c – (a1*b1 + a1*a3*b2+ a2*b2).


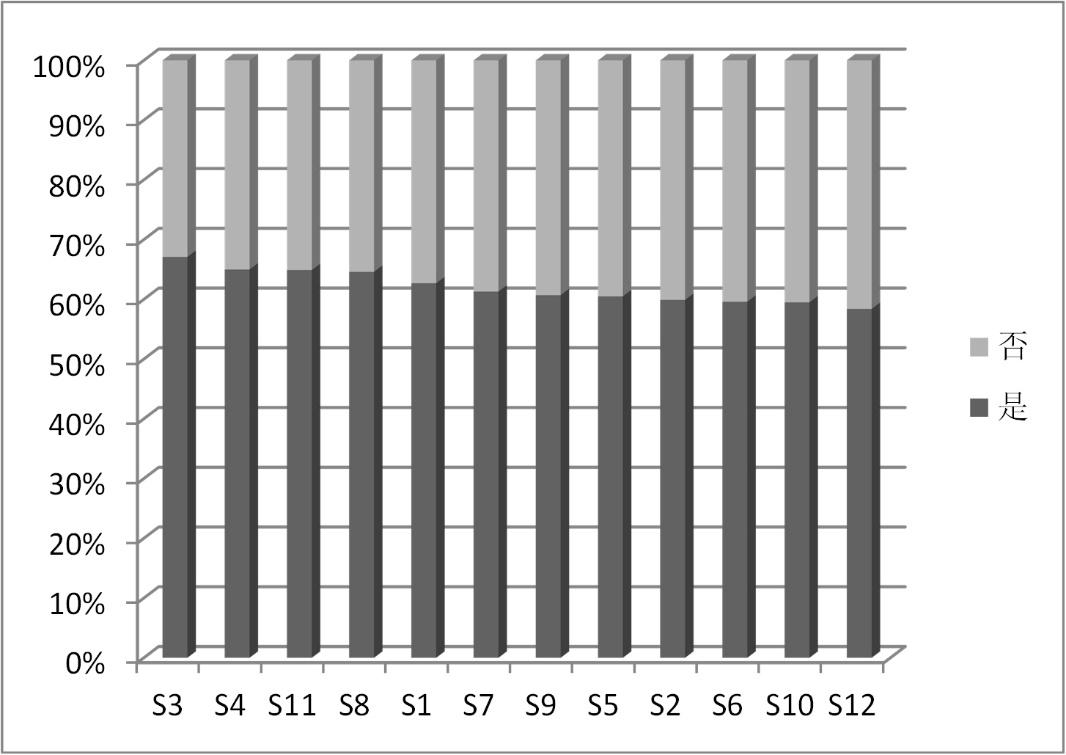


**eFigure 3. Selections of specific Social Support.**


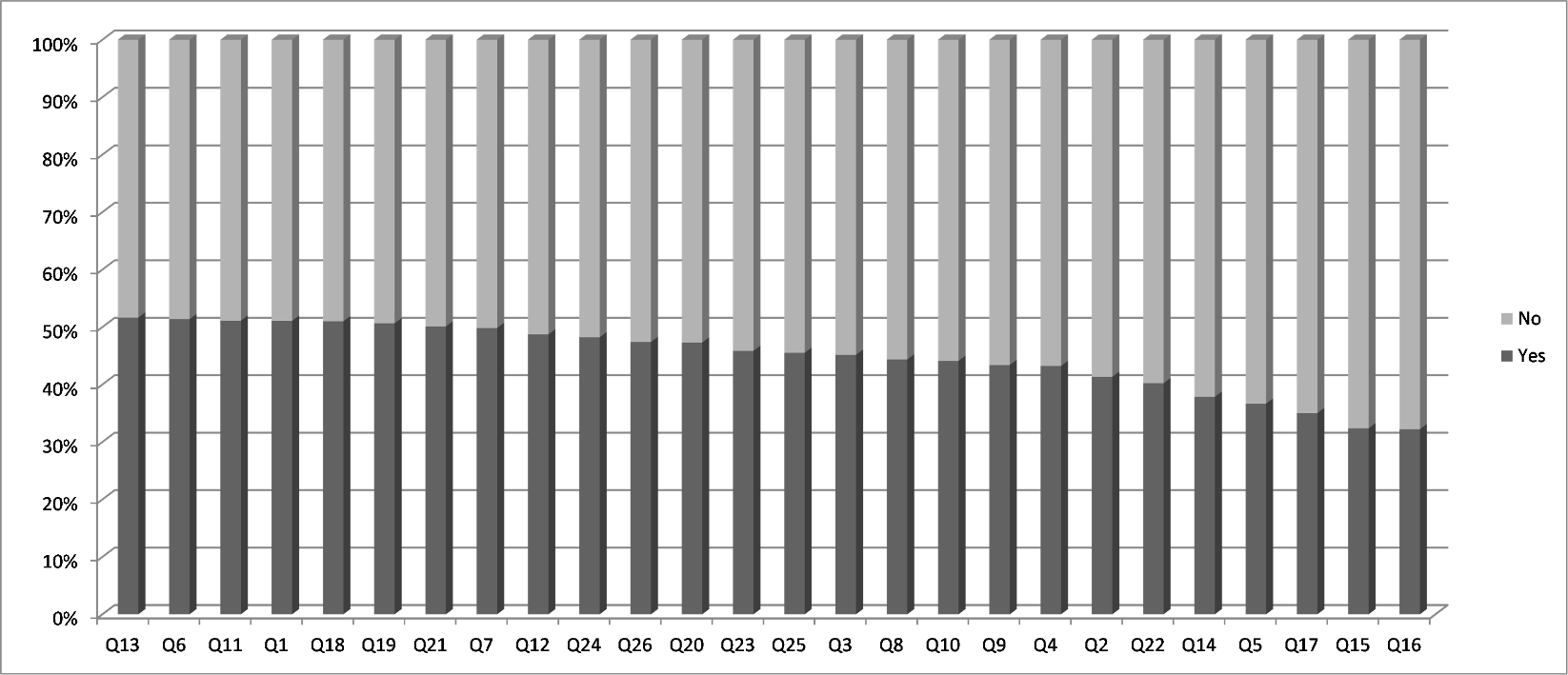


**eFigure 4. Selections of specific self-management items.**

**eTable 1** **Self-management and social support ratings.**

|  |  | *Mean* | Bad | Medium | Good |
| --- | --- | --- | --- | --- | --- |
| **Self-management behavior** |  | 66.32±13.47 | 192(33.62%) | 288(50.44%) | 91(15.94%) |
| SM_A | Diet control management | 66.66±17.32^##^ | 219(38.4%) | 260(45.5%) | 92(16.1%) |
| **SM_B** | Exercise management | **66.50**±17.88**^##^** | **347(60.8%)** | 191(33.5%) | 33(5.8%) |
| SM_C | Medication management | 68.82±17.78^##^ | 207(36.3%) | 146(25.6%) | 218(38.2%) |
| **SM_D** | blood glucose monitoring management | **63.31**±15.90**^##^** | **286(50.1%)** | 174(30.5%) | **111(19.4%)** |
| **SM_E** | Foot Care | **65.79**±15.02**^##^** | **224(39.2%)** | 228(39.9%) | 119(20.8%) |
| **SM_F** | Hypo/hyper glycaemia management | 67.41±16.32^##^ | **233(40.8%)** | 159(27.8%) | 179(31.3%) |
| **Social support** |  |  |  |  |  |
| SS_A | Family support | 14.75±2.99 | **122(21.4%)** | -- | 449(78.6%) |
| SS_B | Friend support | 14.34±2.98 | **137(24.0%)** | -- | 434(76.0%) |
| SS_C | Else support | 14.31±3.01 | **145(25.4%)** | -- | 426(74.6%) |

*Notes*: ^##^ deal with the score index and the standard score, and the value stands for the standard score of each dimensive.

**eTable 2 Correlation matrix and distribution of study variables (Predictor, Mediating, and Criterion Variables, N=571).**

|  | QOL | QOL_A | QOL_B | QOL_C | QOL_D | QOL_E | SM | SM_A | SM_B | SM_C | SM_D | SM_E | SM_F | FPG | SS | SS_A | SS_B | SS_C |
| --- | --- | --- | --- | --- | --- | --- | --- | --- | --- | --- | --- | --- | --- | --- | --- | --- | --- | --- |
| QZ | 1 |  |  |  |  |  |  |  |  |  |  |  |  |  |  |  |  |  |
| SM | **0.369^**^** | 0.344^**^ | 0.307^**^ | 0.333^**^ | 0.284^**^ | 0.286^**^ | 1 |  |  |  |  |  |  |  |  |  |  |  |
| FPG | **-0.451^**^** | -0.394^**^ | -0.405^**^ | -0.395^**^ | -0.345^**^ | -0.378^**^ | **-0.322^**^** | -0.272^**^ | -0.247^**^ | -0.189^**^ | -0.241^**^ | -0.303^**^ | -0.287^**^ | 1 |  |  |  |  |
| SS | **0.390^**^** | 0.342^**^ | 0.330^**^ | 0.355^**^ | 0.297^**^ | 0.330^**^ | **0.323^**^** | 0.370^**^ | 0.248^**^ | 0.229^**^ | 0.177^**^ | 0.237^**^ | 0.240^**^ | **-0.289^**^** | 1 |  |  |  |
| *M* | -29.25 | **-6.36** | **-6.12** | **-8.67** | -4.76 | -3.34 | 86.21^#^ | 20.00^#^ | **13.30**^#^ | **10.32**^#^ | **12.66^#^** | 16.45^#^ | **13.48**^#^ | 7.38 | 43.40 | 14.75 | 14.34 | 14.31 |
| *SD* | 24.41 | 6.84 | 6.21 | 7.62 | 4.45 | 3.32 | 17.51 | 5.20 | 3.58 | 2.67 | 3.18 | 3.75 | 3.26 | 2.51 | 8.41 | 2.99 | 2.98 | 3.01 |

*Note.* SM_A = diet control management, SM_B = exercise management, SM_C = medication management, SM_D = blood glucose monitoring management, SM_E = foot Care, and SM_F = hypo/hyper glycaemia management; QOL_A = activity domain, QOL_B = emotion domain, QOL_C = psychological feeling domain, QOL_D = family burden domain, and QOL_E = diet domain; SS_A = family support, SS_B = friend support, and SS_C = else support; ^#^ stands for the actual total score of each dimension of self-management behavior; ^**^ stands for p < 0.001.

**eTable 3 Regression Results for Mediation (Model 1).**

| Path | B | SE | t | *P* |
| --- | --- | --- | --- | --- |
| a1 | 0.329 | 0.043 | 7.664 | < 0.001 |
| a2 | -0.186 | 0.040 | -4.662 | < 0.001 |
| a3 | -0.260 | 0.043 | -5.995 | < 0.001 |
| b1 | 0.422 | 0.093 | 4.532 | < 0.001 |
| b2 | -0.697 | 0.090 | -7.747 | < 0.001 |
| c’ | 0.496 | 0.085 | 5.834 | < 0.001 |
| c | 0.824 |  |  |  |

*Note*. c=c’+a1b1+a2b2+a1a3b2, ^***^p<0.001.

**etable 4 Bootstrap test results (completely sandardized indirect effects of X on Y).**

| **Mediation path** | **Effect** | **Boot SE** | **P** | **95% CI, lower limit** | **95% CI, upper limit** |
| --- | --- | --- | --- | --- | --- |
| SS→SM→QOL (a1*b1) | 0.0611 | 0.0148 | <0.0001 | 0.0353 | 0.0930 |
| SS→SM→FPG→QOL (a1*a3*b2) | 0.0265 | 0.0063 | <0.0001 | 0.0158 | 0.0421 |
| SS→FPG→QOL (a2*b2) | 0.0665 | 0.0147 | <0.0001 | 0.0406 | 0.0978 |
| SS→QOL (c-c’) | 0.1541 | 0.0198 | <0.0001 | 0.1173 | 0.1953 |

*Abbreviations.* CI = confidence interval; FPG = fasting plasma glucose; SE = standardized estimate; SM = self-management behavior; SS = social support; QOL = quality of life;. *Notes.* SS was the independent variable (X), SM (M1) and FPG (M2) were the mediators, and QOL (Y) was the outcome. a1*b1 and a2*b2 = indirect effects of X on Y through M1 and M2; a1*a3*b2 = indirect effect of X on Y through M1 and M2; and c´ = direct effect of X on Y. The 95% CIs for indirect effects were obtained by bootstrapping with 5,000 resamples. 95% CI, lower limit = lower bound of a 95% CI; 95% CI, upper limit = upper bound of a 95% CI. → = “affects.”

**etable 5 Ratio of indirect to total effect of SS to QOL.**

| **Mediation path** | **Effect proportion** | **Boot SE** | **p** | **95% CI, lower limit** | **95% CI, upper limit** |
| --- | --- | --- | --- | --- | --- |
| SS→SM→QOL (a1*b1) | 0.1569 | 0.0398 | 0.0001 | 0.0899 | 0.2510 |
| SS→SM→FPG→QOL (a1*a3*b2) | 0.0681 | 0.0174 | 0.0001 | 0.0392 | 0.1101 |
| SS→FPG→QOL (a2*b2) | 0.1707 | 0.0384 | <0.0001 | 0.1049 | 0.2523 |
| SS→QOL (c) | 0.3957 | 0.0573 | <0.0001 | 0.2957 | 0.5140 |

*Abbreviations.* CI = confidence interval; FPG = fasting plasma glucose; SE = standardized estimate; SM = self-management behavior; SS = social support; QOL = quality of life;. *Notes.* SS was the independent variable (X), SM (M1) and FPG (M2) were the mediators, and QOL (Y) was the outcome. c = the total effect of X on Y. The 95% CIs for indirect effects were obtained by bootstrapping with 5,000 resamples. 95% CI, lower limit = lower bound of a 95% CI; 95% CI, upper limit = upper bound of a 95% CI. → = “affects.”
